# Prediction error cost exists in the reading processing of Chinese native speakers and advanced Chinese L2 learners

**DOI:** 10.3389/fpsyg.2023.1134229

**Published:** 2023-03-22

**Authors:** Lijuan Feng, Nan Jiang

**Affiliations:** ^1^School of Chinese Language and Literature, University of Chinese Academy of Social Sciences, Beijing, China; ^2^School of Languages, Literatures, and Cultures, University of Maryland, College Park, MD, United States

**Keywords:** context predictability effect, prediction error cost, self-paced reading, lexical prediction, graded prediction

## Abstract

This study applies the paradigm of self-paced reading to examine the Context Predictability Effect in the processing of Chinese and detect whether there is a prediction error cost. Context constraint strength (constraining and neutral) and word predictability (predictable and unpredictable) were strictly manipulated. The statistical results suggest that: (1) There is a Context Predictability Effect for Chinese native speakers in reading processing, which is consistent with most previous studies; (2) There is also a Context Predictability Effect for advanced Chinese L2 learners; (3) Both Chinese native speakers and Chinese L2 learners have a prediction error cost in reading processing, a finding different from those of much previous research. (4) Chinese L2 learners are significantly slower than Chinese native speakers when they conduct predictive reading processing. This paper is very enlightening in that it identifies the existence of a prediction error cost in Chinese L2 processing by means of behavioral experiments, providing evidence for the hypothesis of Lexical Prediction. In a strongly predictive setting, when encountering a plausible but unpredictable word, the brain must expend extra effort to suppress, revise, or reanalyze the material, and this may account for the prediction error cost.

## 1. Introduction

### 1.1. Context predictability effect in L1 processing

Context predictability or context constraint is an important factor affecting the reading processing of words. It refers to the probability that readers predict the target word based on the previous information. The more constrained the context, the fewer words that meet the conditions, and the greater the possibility of the target word being guessed; the weaker the constraint, the more words meet the conditions, the smaller probability of the target word being guessed. This is the Context Predictability Effect (Li et al., 2022).

There is a stable contextual predictive effect in L1 reading processing in both Western languages and Chinese (Zhu, 1991; Bai et al., 2011; Roland et al., 2012; Staub, 2015; Kwon et al., 2017; Chow et al., 2018; Zhang et al., 2019, 2023; Zhao, 2022). In eye movement studies, the constraint of the context can affect the target word fixation time: the more constrained the context is, the shorter the fixation time will be; the less constrained the context, the longer the fixation time (Rayner and Well, 1996; Rayner et al., 2004, 2005, 2006). Rayner and Well (1996) divided contexts into three types, high-constraint, medium-constraint, and low-constraint, and investigated the influence of context constraint on eye movement in reading. It was found that the fixation time of participants in the low-constraint context was longer than that in the high-constraint and medium-constraint contexts. Rayner et al. (2005) used a Chinese corpus as the research object and copied the research process of Rayner and Well (1996), and the results were highly consistent with Rayner and Well (1996). In addition, word skipping is more likely to occur in high- contexts than in low-constraint contexts (Rayner et al., 2005; Bai et al., 2008; Guo, 2012; Zhang, 2015; Li, 2016). This phenomenon also exists among child readers. Children under the high-constraint context had greater skipping rates and spent less time reading (Zhao, 2020).

There are also many studies on the interaction between context predictability and other factors, as follows: Liu et al. (2020) explored how context predictability affects Chinese vocabulary processing in reading by observing the interaction between context predictability, whole word frequency, and Chinese character frequency. The results showed that context predictability, whole word frequency, and first character frequency affect vocabulary processing relatively independently; contextual predictability directly affects the processing of the second character within words. Liu et al. (2019) divided children’s reading skills into high and low groups and used the boundary paradigm, which changes with fixation, to investigate the impact of context predictability on children’s parafoveal processing. The results showed that children with high reading skills had earlier use of context predictability than children with low reading skills. Word frequency and context predictability also have an impact on the reading of the elderly (Wang et al., 2012), who adopt more cautious reading strategies when the difficulty of reading content is too high or too low. Under the right conditions, older adults rely more heavily on contextual predictive information than younger adults (Zhao, 2009).

In summary, the context predictability effect is mainly manifested in the promotion of target word processing by high-constraint contexts; that is, in high-constraint contexts, readers process target words faster and with lower processing difficulty (Wang, 2017; Zhao et al., 2021). Conversely, if an unpredictable word appears instead of a predictable word in a high-constraint context, will the reader’s processing be disturbed? In other words, will there be a prediction error cost? This is another issue we are concerned about.

### 1.2. Prediction error cost in L1 processing

At present, researchers have not yet reached a consensus on whether the prediction error cost exists. There are two main views. One view is that there is a prediction error cost (Coulson and Van Petten, 2002; Moreno et al., 2002; Wicha et al., 2004; DeLong et al., 2005, 2007, 2011, 2012; Van Berkum et al., 2005; Federmeier et al., 2007). This view holds that in a high-constraint context, the specific vocabulary with the highest cloze score is activated first, and when the reader encounters a word other than the target word in the reading process, it will violate the previous prediction, resulting in processing interference. This is the Lexical Prediction view. Another view is that there is no prediction error cost (Luke and Christianson, 2016; Frisson et al., 2017; Zhao et al., 2021; Zhao, 2022). This view holds that the reader does not predict a specific word in reading processing, but activates a series of words in parallel to varying degrees. This is the Graded Prediction view.

Luke and Christianson (2016) used a method of large-scale survey to explore the role of context in reading processing. The study did not find a prediction error cost and believed that the high-constraint context activates not a whole word, but more semantic and morphosyntactic information related to it, thus supporting the Graded Prediction view. Frisson et al. (2017) used the method of controlled experimental design for the first time to provide evidence for the conclusion of Luke and Christianson (2016). The experiment adopts a design of 2 (context constraint strength: constraining and neutral) * 2 (word predictability: predictable and unpredictable). The context constraint strength is one variable and this variable has two levels, which are high-constraint context and low-constraint context; another variable is lexical predictability, which also has two levels, predictable words, and unpredictable words. The predictable words here are the specific words that have the greatest possibility of being predicted in the high-prediction context we discussed above, and the unpredictable words are other words that cannot be predicted in advance but reasonable semantically. The innovation of this study is to judge whether there is a prediction error cost by comparing the processing time of unpredictable words in high-constraint contexts and low-constraint (neutral) contexts. The basic logic is: if there is a prediction error effect, then the processing difficulty of unpredictable words in a high-constraint context must be greater than that in a low-constraint context, and correspondingly more processing time will be spent. The experiment did not find a prediction error cost, supporting the view of Graded Prediction.

We believe that one of the reasons why the prediction error cost was not found may be that the constraint of the high-constraint context is not enough, Frisson et al. (2017) is only 70.2%, Zhao et al. (2021) improved this problem by increasing the constraint in the high-constraint context, but still found no prediction error effect. The key point is that the eye movement data of first fixation time and skipping rate in Zhao et al. (2021) reflect the situation of early parafoveal processing, while the results of ERP research on prediction error cost indicated that the interference on the processing of unpredictable words in a high-constraint context is likely to occur in the later stage (Moreno et al., 2002; Federmeier, 2007; Federmeier et al., 2007; Delong et al., 2011; DeLong et al., 2012).

### 1.3. Context predictability effect in L2 processing

On whether there is a context predictability effect in second language reading processing, researchers have not yet reached a consensus. Some studies have shown that even at an advanced level, L2 learners cannot predict information during reading processing, at least not to the same extent as native speakers (Kaan et al., 2010; Grüter and Rohde, 2013; Kaan, 2014). The difference in grammatical processing between L2 learners and native speakers is mainly affected by the following factors: incomplete acquisition of the target grammar, cognitive limitation of the target language, interference from the grammar and processing system of first language, etc. Other studies suggest that L2 learners can make predictions just like native speakers (Clahsen and Felser, 2006; Chambers and Cooke, 2009; Dussias et al., 2013). In Spanish grammar, there is a distinction between feminine and masculine nouns, and the modifiers should be consistent in gender with the nouns. Dussias et al. (2013) took advantage of this characteristic of Spanish grammar to investigate whether L2 learners can activate nouns through the gender marker of the modifiers. The results showed that advanced L2 learners of English showed the same context predictability effect as native speakers.

### 1.4. Prediction error cost in L2 processing

We only found one article on prediction error cost in L2 processing. Zirnstein et al. (2018) used ERP experiments to prove that L2 learners can not only predict upcoming words through context but also generate prediction error cost like native speakers. The performance of L2 learners in processing unpredictable words is related to their mastery of native language regulations. L2 learners with a better grasp of native language regulations have larger frontal positivity when processing unpredictable words, and this effect is attenuated by cognitive control, especially inhibitory control ability. Inhibition control ability appears to mediate the difficulty readers incur.

### 1.5. Current study

We took Chinese native speakers and advanced Chinese L2 learners as research objects, used the method of self-paced reading, and replicated the experimental design of Frisson et al. (2017). The following are research questions: 1. Are there context predictability effect and prediction error cost in the reading processing for Chinese native speakers? 2. Are there context predictability effect and prediction error cost in the reading processing for advanced Chinese L2 learners? 3. Is there any difference in this regard between Chinese native speakers and advanced Chinese L2 learners? Our experimental design has made the following improvements: 1. We improved the constraint strength of high-constraint contexts based on Frisson et al. (2017); 2. We used the method of self-paced reading, because this method is closest to natural reading (Mitchell and Green, 1978, p. 610); 3. We examined the potential differences of prediction mechanism between Chinese native speakers and Chinese L2 learners. Doing so can provide theoretical support and guidance for the teaching of Chinese as a second language.

## 2. Experiment 1

### 2.1. Method

#### 2.1.1. Participants

Twenty six participants (10 males) were recruited from a university in Hebei, China, aged 18–34 (*M* = 20.30, SD = 3.08). These participants are all Chinese speaking, have undergone at least some undergraduate education, and have normal vision or corrected vision.

#### 2.1.2. Design

Experiment 1 adopted a two-factor within-subjects design, which is 2 (context constraint strength: constraining context and neutral context) * 2 (word predictability: predictable word and unpredictable word). As shown in Table 1.

**Table 1:** Sample items in Experiment 1.

Conditions	Example
Constraining context- Predictable word (CP)	大卫送给妈妈的生日礼物让妈妈特别感动。 David’s birthday gift to her mother especially touched her.
Constraining context- Unpredictable word (CU)	大卫送给妈妈的生日卡片让妈妈特别感动。 David’s birthday card to her mother especially touched her.
Neutral context- Predictable word (NP)	我看到这个礼物的时候非常惊讶。 When I saw this gift, I was very surprised.
Neutral context- Unpredictable word (NU)	我看到这个卡片的时候非常惊讶。 When I saw this card, I was very surprised.

The target word is underlined.

#### 2.1.3. Materials

We selected 106 contexts from Chinese textbooks at junior and intermediate level, including 53 high-constraint and 53 low-constraint contexts. Each context contains one predictable word and one unpredictable word. For example: “大卫送给妈妈的生日礼物/卡片让妈妈特别感动 (David’s birthday gift/card to her mother especially touched her)” is a high-constraint context. In this high-constraint context, “礼物 (gift)” is a predictable word, and “卡片 (card)” is an unpredictable word; “我看到这个礼物/卡片的时候非常惊讶 (When I saw this gift/card, I was very surprised)” is a low-constraint context that also contains the words “礼物 (gift)” and “卡片 (card).” To avoid the interference of the packing effect at the end of the sentence, the position of the target word is set in the middle of each sentence.

First, we removed the target words from 53 high-constraint contexts and asked 31 native Chinese speakers to fill in the blanks with the first word that comes to their mind. In previous studies, the entire sentence was presented at once. But in the self-paced experiment, the vocabulary is presented sequentially, and the participants can only predict through the context before the target word. To match the real reading process as much as possible, we only presented the content before the blanks. After getting the cloze data, we selected the word given by the largest number of people as the predictable word. The ratio of the number of people giving the target word to the total number of people is called the constraint strength (cloze value). We deleted the high-constraint contexts with a constraint strength below 60%, and the average constraint strength reached 82.4%. We finally retained 37 high-constraint contexts, plus 37 low-constraint contexts, for a total of 74 contexts. Secondly, the 74 contexts were divided into 148 sentences, which were divided into two groups by means of Latin squares. Then, 29 and 41 native Chinese speakers were invited to judge the semantic rationality of the sentences, using the Likert scale. Sentences with an average value above 4 were selected, and a total of 128 target sentences were obtained.

The target sentences were divided into 4 groups. The constraint strength of constraining context with predictable word (CP) is 82.4%, that of constraining context with unpredictable word (CU) is 3.7%, and that of neutral context with predictable word (NP) is 2.4%, while neutral context with unpredictable word (NU) is 2.3%. The results of paired-sample *t*-tests showed that constraint strength of CP was significantly higher than that of CU, *t*(31) = −33.114, *p* < 0.001, and NP, *t*(31) = −35.065, *p* < 0.001; there was no significant difference between CU and NU, *t*(31) = 1.603, *p* = 0.119. We balanced character frequency and number of strokes of predictable and unpredictable words from the first character to the fifth character, first character frequency: *t*(31) = 0.051, *p* = 0.960, first character stroke number: *t*(31) = −0.651, *p* = 0.520; second character frequency: *t*(31) = 1.006, *p* = 0.322, second character number of strokes: *t*(31) = 0.896, *p* = 0.377; third character frequency: *t*(31) = 0.424, *p* = 0.674, third character number of strokes: *t*(31) = 0.056, *p* = 0.956; fourth character frequency: *t*(31) = −0.303, *p* = 0.764, fourth character number of strokes: *t*(31) = 0.823, *p* = 0.417; and fifth character frequency: *t*(31) = −1.657, *p* = 0.108, fifth character number of strokes: *t*(31) = −0.113, *p* = 0.911. Character frequency statistics are based on SUBTLEX-CH (Cai and Brysbaert, 2010), and the unit is times/million. In the self-paced reading experiment, we also added 49 semantically unreasonable sentences as distractors.

#### 2.1.4. Procedure

**Figure 1:**
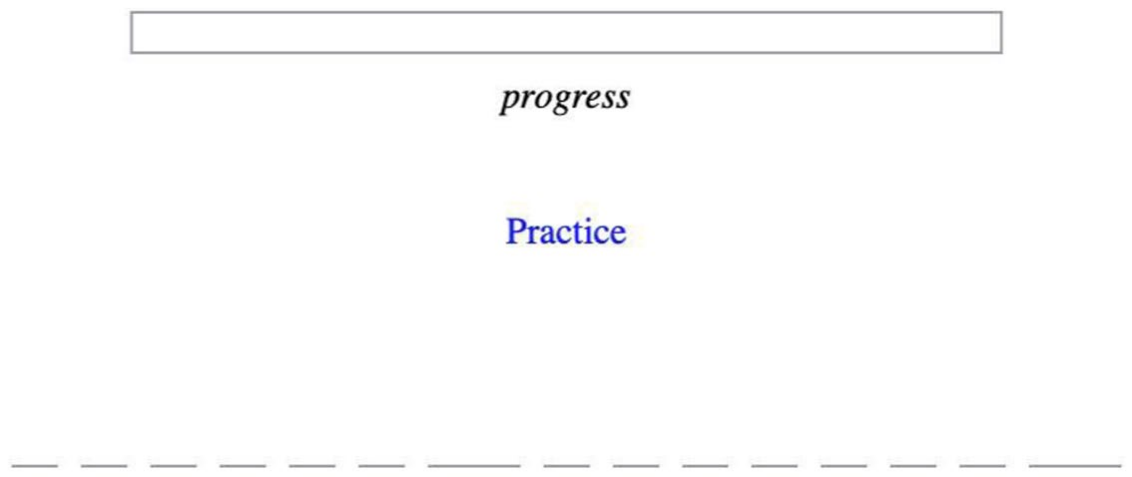
Practice page of the formal experiment.

We set up the self-paced reading experiment at.^1^ The system automatically divided the experimental materials into two groups according to the Latin square and randomly assigned them to the participants. Using the online experiment method can make participants more relaxed and get closer to the state of natural reading. Before the experiment, participants were required to prepare a computer in an environment with normal network speed. After accessing the webpage, participants saw a brief description of the experiment and needed to fill in information such as mother tongue, age, years of learning Chinese, gender, and email address first; then, they were required to finish the experiment as quickly and as well as possible. The first page of the formal experiment is as shown in Figure 1.

The participant should press the space bar of the computer to start reading. Sentences are presented in units of Chinese characters, from left to right; participants press the space bar once to make the next Chinese character appear. After they finish reading a sentence, participants are required to answer a multiple-choice question. There are 6 practice sentences, and the target materials start from the seventh sentence. Each participant needs to read 113 sentences (64 target sentences, 49 distractor items), and the experiment takes 15–20 min in total. The system records the reaction time for each Chinese character and the correct rate of answers.

We did not present word by word for the following reasons: First, Chinese words vary in length. In Chinese, although two-syllable words are dominant, there are still many monosyllabic words, such as “是 (is),” and three-syllable words, such as “有时候 (sometimes),” and even words with more syllables. Therefore, the reaction time for given words is affected by their number of characters. Second, word-by-word presentation is not conducive to the analysis of the spillover effect. We analyzed the reaction time for three more Chinese characters after the target word; if stimuli were presented word by word, the spillover effect was not easy to measure. Third, character-by-character presentation is the closest to the typesetting of authentic reading materials in Chinese.

### 2.2. Results

The correct rate of the answers was above 90%, which proved that the participants had read the sentences carefully. We recorded the reaction time for the target word, which contained two characters, and the three characters following it. We deleted the data according to the following criteria: (1) if the correct rate of a participant is less than 60%, delete all the data for that participant; (2) data whose reaction time is shorter than 80 ms but greater than 1,500 ms; (3) data beyond ±3 standard deviations. A total of 492 data points were deleted, accounting for 4.6% of the total data.

We constructed linear mixed models of the self-paced reading data, using the lme4 package (Bates et al., 2015) in R version 4.2.2. We mainly examine the responses in five regions: the first character of the target word, the second character of the target word, and the third, fourth and fifth characters after that. The extra three characters after the target word are examined because there is a spillover effect in the self-paced experiment.

**Figure 2:**
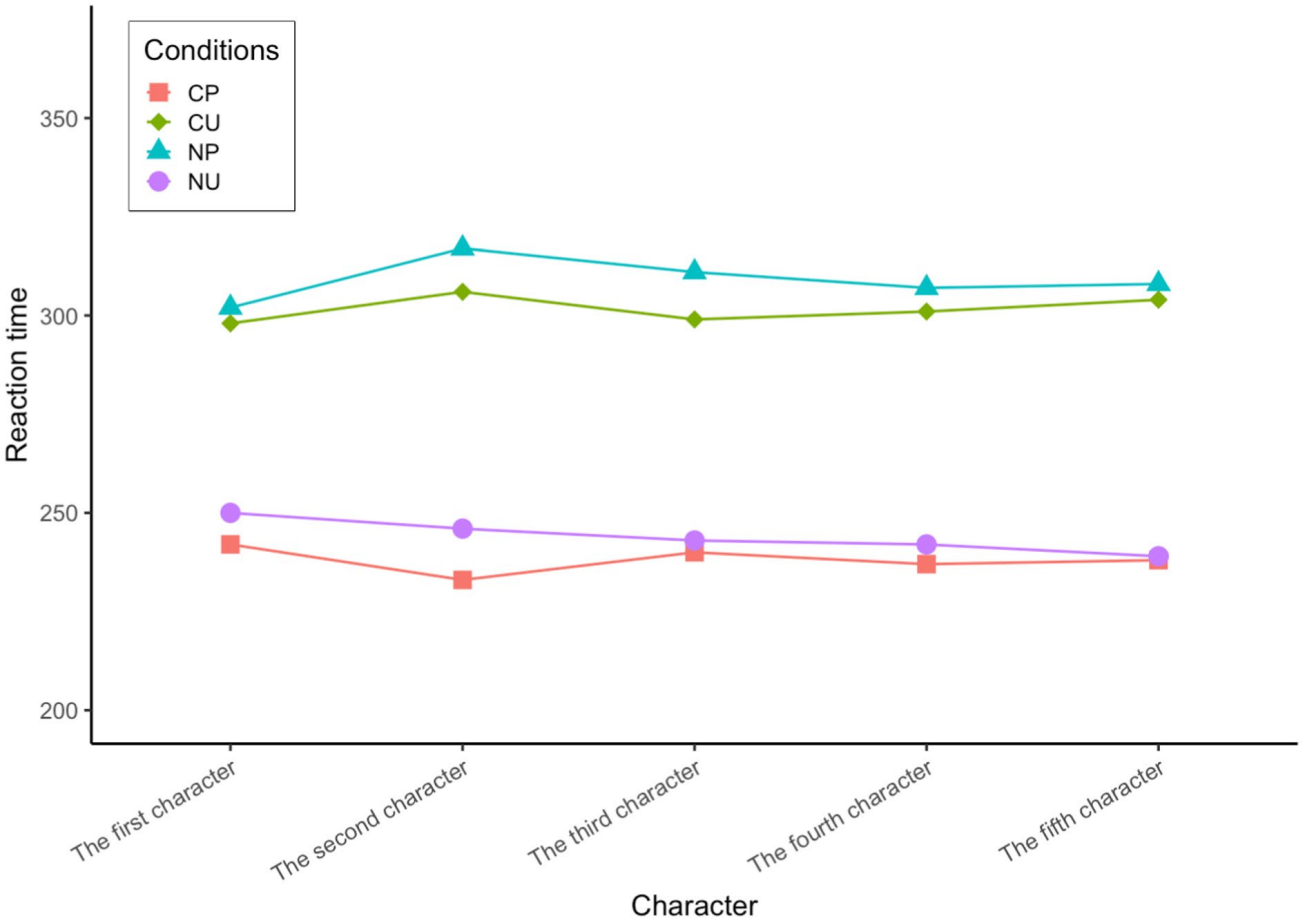
Reaction time of Chinese native speakers in four conditions.

There was a significant difference in reaction time from the first character to the fifth character between the conditions of CP and NP: first character: *t*(223) = −3.967, *p* < 0.01; second character: *t*(347) = −4.859, *p* < 0.01; third character: *t*(348) = −5.128, *p* < 0.01; fourth character: *t*(345) = −5.300, *p* < 0.01; fifth character: *t*(347) = −5.609, *p* < 0.01, and CP < NP (see Figure 2). This shows that processing time for predictable words in high-constraint contexts is significantly shorter than that in low-constraint contexts. There was also a significant difference in reaction time from the first character to the fifth character between the conditions of CP and CU, first character: *t*(223) = −3.788, *p* < 0.01; second: *t*(346) = −3.832, *p* < 0.01; third: *t*(350) = −4.222, *p* < 0.01; fourth: *t*(345) = −4.753, *p* < 0.01; fifth: *t*(345) = −5.112, *p* < 0.01, and CP < CU (see Figure 2). This suggests that in high-constraint contexts, processing time for predictable words is significantly shorter than that for unpredictable words. The above two points confirm the existence of the context predictability effect in Chinese native speakers’ reading processing.

There was a significant difference in reaction time from the first character to the fifth character between the conditions of CU and NU, first character: *t*(221) = 3.145, *p* < 0.05; second character: *t*(349) = 3.439, *p* < 0.05; third character: *t*(349) = 4.014, *p* < 0.05; fourth character: *t*(344) = 4.448, *p* < 0.01; fifth character: *t*(344) = 5.007, *p* < 0.01, and CU > NU (see Figure 2). The processing time of unpredictable words in the high-constraint context is significantly longer than that in low-constraint context, indicating that there is a prediction error cost. Descriptive statistics are presented in Table 2.

**Table 2:** The average reaction time (in milliseconds) and standard error of Chinese L1 speakers.

Conditions	The first character	The second character	The third character	The fourth character	The fifth character
CP	242 (132.70)	233 (122.19)	240 (135.01)	237 (128.37)	238 (134.01)
NP	302 (71.64)	317 (94.64)	311 (71.34)	307 (71.09)	308 (70.94)
CU	298 (63.71)	306 (87.54)	299 (68.21)	301 (66.56)	304 (70.22)
NU	250 (147.87)	246 (140.97)	243 (133.88)	242 (133.99)	239 (133.12)

## 3. Experiment 2

### 3.1. Method

#### 3.1.1. Participants

A total of 19 Chinese L2 learners (9 males), aged 18–34 (M = 23.15, SD = 5.39), were recruited from the fourth grade (of five grades) of the Chinese summer program of a university in the United States. All of them were English native speakers. The participants had been learning Chinese for more than 3 years and can be assessed as advanced-level learners. They were able to read the experimental material with no difficulty, for two reasons: 1. The experimental materials were selected from Chinese teaching textbooks at junior and intermediate level; 2. We asked two Chinese L2 learners from the same group with participants to finish a pre-test to ensure that no unfamiliar words appeared.

#### 3.1.2. Design

The experimental design was identical to that in Experiment 1.

#### 3.1.3. Materials

The experimental materials were identical to those in Experiment 1.

#### 3.1.4. Procedure

The experimental procedure was identical to that in Experiment 1.

### 3.2. Results

The correct rate of the questions was above 85%, which proved that the participants read the sentences carefully. We recorded the reaction time of the target word, which contained two characters, and the three characters following it. We deleted data according to the following criteria: (1) Data whose reaction time was shorter than 80 ms or greater than 1,500 ms; (3) Data beyond ±3 standard deviations. A total of 226 data points were deleted, accounting for 7.4% of the total data.

We constructed linear mixed models of the self-paced reading data, using the lme4 package (Bates et al., 2015) in R version 4.2.2 (R Core Team, 2022). We mainly examine the responses of five regions: the first character of the target word, the second character of the target word, and the third, fourth and fifth characters after that.

**Figure 3:**
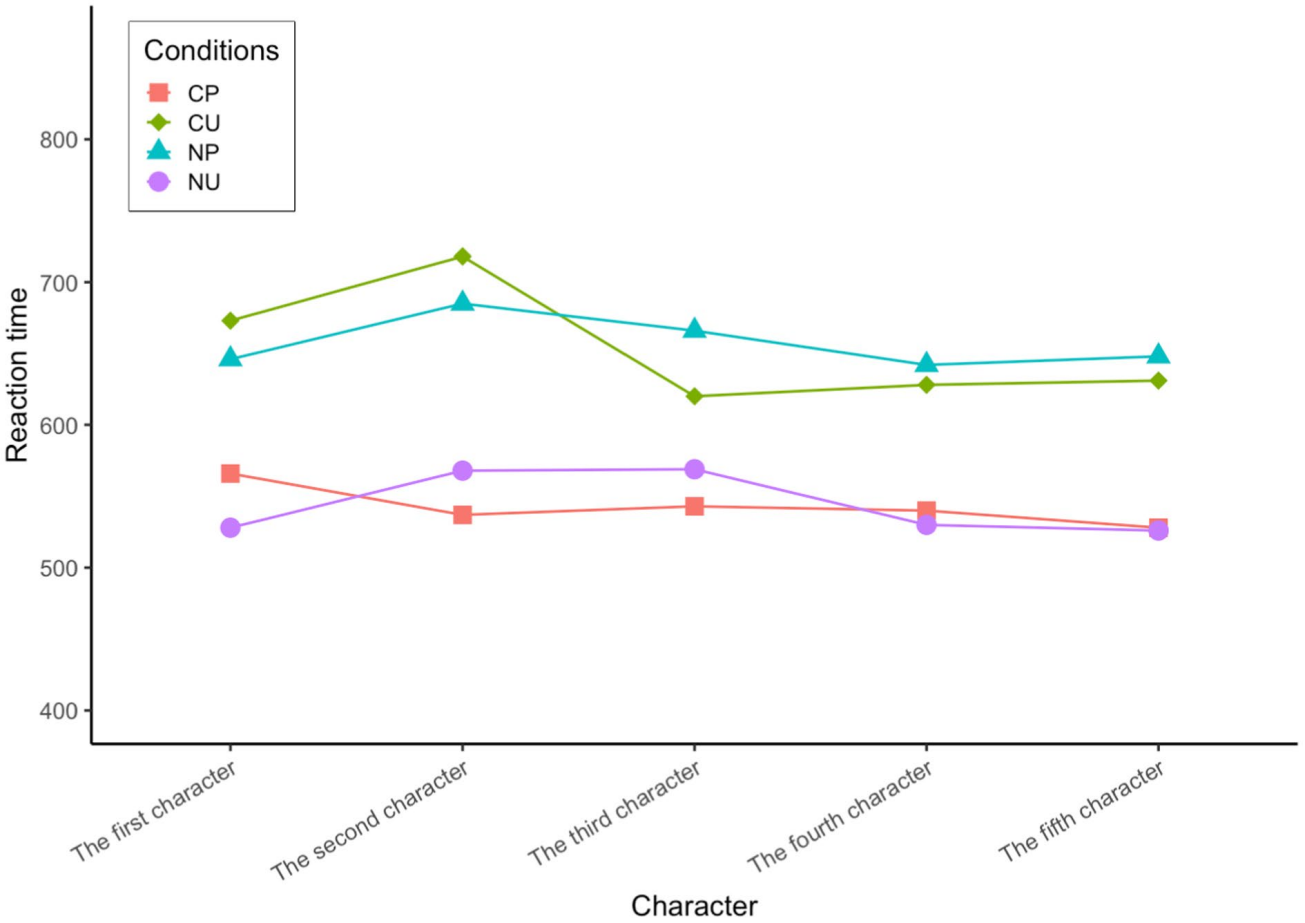
Reaction time of Chinese L2 Learners in four conditions.

There was a significant difference in the reaction time from the first character to the fifth character between the conditions of CP and NP, first character: *t*(392) = −6.440, *p* < 0.01; second character: *t*(606) = −6.388, *p* < 0.01; third character: *t*(581) = −6.524, *p* < 0.01; fourth character: *t*(572) = −5.933, *p* < 0.01; fifth character: *t*(584) = −6.612, *p* < 0.01, and CP < NP (see Figure 3). This shows that the processing time of predictable words in high-constraint contexts is significantly shorter than that in low-constraint contexts. There was also a significant difference in the reaction time from the first to the fifth character between the conditions of CP and CU, the first character: *t*(389) = −6.152, *p* < 0.01; the second character: *t*(588) = −7.539, *p* < 0.01; the third character: *t*(587) = −4.676, *p* < 0.01; the fourth character: *t*(600) = −5.383, *p* < 0.01; the fifth character: *t*(580) = −6.311, *p* < 0.01, and CP < CU (see Figure 3). This shows that in high-constraint contexts, the processing time of predictable words is significantly shorter than that of unpredictable words. The above two points confirm the existence of a context predictability effect in Chinese L2 learners’ reading processing.

There was also a significant difference in the reaction time for the first, second, fourth and fifth characters between the conditions of CU and NU, the first character: *t*(386) = 5.912, *p* < 0.01; the second character: *t*(608) = 22.712, *p* < 0.01; the third character: *t*(585) = 3.664, *p* < 0.01; the fourth character: *t*(596) = 5.902, *p* < 0.01; the fifth character: *t*(575) = 6.070, *p* < 0.01, and CU > NU (see Figure 3). The processing time of unpredictable words in high-constraint context is significantly longer than that in low-constraint context, indicating that there is a prediction error cost. Descriptive statistics are as follows (see Table 3)

**Table 3:** The average reaction time (in milliseconds) and standard error of Chinese L2 learners.

Conditions	The first character	The second character	The third character	The fourth character	The fifth character
CP	566 (195.88)	537 (205.49)	543 (210.18)	540 (203.24)	528 (196.48)
NP	646 (182.27)	685 (174.89)	666 (153.96)	642 (167.26)	648 (183.65)
CU	673 (199.38)	718 (198.23)	620 (158.43)	628 (157.89)	631 (148.79)
NU	528 (183.28)	568 (199.04)	569 (208.89)	530 (201.54)	526 (189.09)

## 4. Comparison between two experimental results

We compared data from Chinese native speakers and Chinese L2 learners. There was a significant difference in the reaction time for all the conditions. For the first character, CP: *t*(218) = 20.891, *p* < 0.01; CU: *t*(420) = 20.837, *p* < 0.01; NP: *t*(424) = 20.937, *p* < 0.01; NU: *t*(214) = 20.633, *p* < 0.01. For the second character, CP: *t*(2412) = 18.408, *p* < 0.01; CU: *t*(604) = 19.808, *p* < 0.01; NP: *t*(624) = 17.672, *p* < 0.01; NU: *t*(2411) = 19.819, *p* < 0.01. For the third character, CP: *t*(2408) = 23.043, *p* < 0.01; CU: *t*(602) = 20.642, *p* < 0.01; NP: *t*(594) = 21.790, *p* < 0.01; NU: *t*(2404) = 24.046, *p* < 0.01. For the fourth character, CP: *t*(2413) = 23.393, *p* < 0.01; CU: *t*(608) = 21.214, *p* < 0.01; NP: *t*(581) = 21.528, *p* < 0.01; NU: *t*(2402) = 22.597, *p* < 0.01. This indicates that advanced Chinese L2 learners were significantly slower than Chinese native speakers when they did predictive processing. In a word, even though advanced Chinese L2 learners can establish the same prediction mechanism as Chinese native speakers, there is still a big gap in terms of speed of predictive reading between them.

## 5. Discussion

In response to the questions raised above, our results are: There is a context predictability effect in the reading processing of Chinese L1, which is consistent with the current academic consensus. There is a prediction error cost in reading processing for Chinese L1 speakers. The ERP result of Federmeier et al. (2007), DeLong et al. (2011, 2012), Chou et al. (2014) was proved by our behavioral experiments. There is a context predictability effect in the reading processing of advanced Chinese L2 learners. This is consistent with the findings of Clahsen and Felser (2006), Chambers and Cooke (2009), and Dussias et al. (2013). There is a prediction error cost in reading processing for advanced Chinese L2 learners. The ERP result of Zirnstein et al. (2018) is supported by our behavioral experiments. These results suggest that Chinese L2 learners can establish the same prediction mechanism as Chinese native speakers in reading processing.

The brain seems to cope with the speed and complexity of language processing by “thinking ahead,” that is, generating information about what might come and preparing to process it in advance at multiple levels, and this predictive processing brings benefit; at the same time, it will also bring cost (Federmeier, 2007). The benefit is manifested in the facilitation of the context predictability effect in L1 and L2 processing; that is, the processing time of predictable words is shorter than that of unpredictable words in high-constraint contexts (CP < CU) and that of predictable words in a high-constraint context is shorter than that in a low-constraint context (CP < NP); the cost is manifested in that the brain takes longer to process unpredictable words, because early activated target words are replaced, thus causing processing interference (CU > NU).

We can provide explanations for the above conclusions from two perspectives. One is Lexical Prediction. This theory holds that in the high-constraint context, target word activation is an “all or nothing” serial approach (DeLong et al., 2014; Kwon et al., 2017), so that when what is activated is not the target word, the brain will enter a “nothing” mode, that is, it needs to use more cognitive space to deal with emergencies, resulting in a prediction error cost.

The second explanation is Frontal LP (Late Positivity) Effects. Federmeier et al. (2007) observed the ERP data of the participants in the two intervals of 300–500 ms and 500–900 ms when they processed the unpredictable word in strongly constraining context and found the increased Frontal Positivity from 500 to 900 ms post-stimulus-onset, namely frontal LP effects. This discovery is very critical. On the one hand, it provides a possible explanation for the existence of prediction error cost, and on the other hand, it provides a new methodology for the research on contextual constraints. Delong et al. (2011) proved the existence of frontal LP effects, and further proposed that this effect is likely to occurs in parallel with the N400 of semantic activation and integration.

The reason why it is called processing “cost” is that when encountering an unpredictable component, the brain may need to invest more “extra” processing to override, revise, inhibit, or reanalyze the accruing contextual representation (Kutas et al., 2011; DeLong et al., 2012; Chou et al., 2014). For learners, in the short term, it may be “cost,” in the long run, it seems that readers will benefit from such a process (Kutas et al., 2011).

In addition, cognitive control ability also plays an important role in reading processing, especially inhibitory control. When readers encounter processing difficulties, meaning that their predictions are denied, they can play a mediating role. The cost of L2 reading is higher for L2 learners if they have poorer inhibitory control, and the same pattern of effect was found for L1 speakers (Zirnstein et al., 2018). Therefore, in second language teaching and learning, consciously training learners’ cognitive control, especially inhibitory control ability, is an effective way to improve reading ability.

## 6. Conclusion

This paper proves the existence of context predictability effect and prediction error cost in the reading processing of Chinese native speakers and advanced Chinese L2 learners. At an advanced level, Chinese L2 learners can build the same prediction mechanism as Chinese native speakers, but the learners are significantly slower than the native speakers in predictive riding processing. Teachers can take advantage of this to train Chinese L2 learners to consciously use predictive strategies, thereby improving reading ability and speed. The experimental results can be explained by Lexical Prediction and Frontal LP Effects.

## Data availability statement

The raw data supporting the conclusions of this article will be made available by the authors, without undue reservation.

## Author contributions

LF is responsible for the specific content of the paper, including the writing of the literature review, the implementation of experiments, and data statistics, etc. NJ guides the paper in terms of theory and methods. All authors contributed to the article and approved the submitted version.

## Conflict of interest

The authors declare that the research was conducted in the absence of any commercial or financial relationships that could be construed as a potential conflict of interest.

## Publisher’s note

All claims expressed in this article are solely those of the authors and do not necessarily represent those of their affiliated organizations, or those of the publisher, the editors and the reviewers. Any product that may be evaluated in this article, or claim that may be made by its manufacturer, is not guaranteed or endorsed by the publisher.

## Footnotes

^1^ https://spellout.net/ibexfarm

## References

[ref1] Bai X. J. Cao Y. X. Gu J. J. Guo Z. Y. Y G. L. (2011). Effect of p predictability and space on Chinese reading: an eye movement study. J. Psychol. Sci. 34, 1282–1288. doi: 10.16719/j.cnki.1671-6981.2011.06.021

[ref2] Bai X. J. Liu L. P. Yan G. L. (2008). An eye movement study on word-skipping in sentence Reading. J. Psychol. Sci. 31, 1045–1048. doi: 10.16719/j.cnki.1671-6981.2008.05.005

[ref4] Bates D. Maechler M. Bolker B. Walker S. Christensen R. H. Singmann H.. (2015). lme4: Linear mixed-effects models using Eigen and S4. R package version 1, 2014.

[ref5] Cai Q. Brysbaert M. (2010). SUBTLEX-CH: Chinese word and character frequencies based on film subtitles. PLoS One 5:e10729. doi: 10.1371/journal.pone.0010729, PMID: 20532192 PMC2880003

[ref6] Chambers C. G. Cooke H. (2009). Lexical competition during second-language listening: sentence context, but not proficiency, constrains interference from the native lexicon. J. Exp. Psychol. Learn. Mem. Cogn. 35, 1029–1040. doi: 10.1037/a0015901, PMID: 19586268

[ref7] Chou C. J. Huang H. W. Lee C. L. Lee C. Y. (2014). Effects of semantic constraint and cloze probability on Chinese classifier-noun agreement. J. Neurolinguistics 31, 42–54. doi: 10.1016/j.jneuroling.2014.06.003

[ref8] Chow W. Y. Lau E. Wang S. Phillips C. (2018). Wait a second! Delayed impact of argument roles on on-line verb prediction. Lang. Cogn. Neurosci. 33, 803–828. doi: 10.1080/23273798.2018.1427878

[ref9] Clahsen H. Felser C. (2006). Grammatical processing in language learners. Appl. Psycholinguist. 27, 3–42. doi: 10.1017/S0142716406060024 19337839

[ref10] Coulson S. Van Petten C. (2002). Conceptual integration and metaphor: an event-related potential study. Mem. Cogn. 30, 958–968. doi: 10.3758/BF03195780, PMID: 12450098

[ref11] DeLong K. A. Groppe D. M. Urbach T. P. Kutas M. (2012). Thinking ahead or not? Natural aging and anticipation during reading. Brain Lang. 121, 226–239. doi: 10.1016/j.bandl.2012.02.006, PMID: 22406351 PMC3571658

[ref12] DeLong K. A. Troyer M. Kutas M. (2014). Pre-processing in sentence comprehension: sensitivity to likely upcoming meaning and structure. Lang. Linguis. Compass 8, 631–645. doi: 10.1111/lnc3.12093, PMID: 27525035 PMC4982702

[ref13] Delong K. A. Urbach T. P. Groppe D. M. Kutas M. (2011). Overlapping dual ERP responses to low cloze probability sentence continuations. Psychophysiology 48, 1203–1207. doi: 10.1111/j.1469-8986.2011.01199.x, PMID: 21457275 PMC3131420

[ref14] DeLong K. A. Urbach T. P. Kutas M. (2005). Probabilistic word pre-activation during language comprehension inferred from electrical brain activity. Nat. Neurosci. 8, 1117–1121. doi: 10.1038/nn1504, PMID: 16007080

[ref15] DeLong K. A. Urbach T. P. Kutas M. (2007). A cost to mispredicting: effects of sentential constraint violations. Poster presented at the 20th Annual CUNY Conference on Human Sentence Processing La Jolla, CA.

[ref16] Dussias P. E. Kroff J. R. V. Tamargo R. E. G. Gerfen C. (2013). When gender and looking go hand in hand: grammatical gender processing in L2 Spanish. Stud. Second. Lang. Acquis. 35, 353–387. doi: 10.1017/S0272263112000915

[ref17] Federmeier K. D. (2007). Thinking ahead: the role and roots of prediction in language comprehension. Psychophysiology 44, 491–505. doi: 10.1111/j.1469-8986.2007.00531.x, PMID: 17521377 PMC2712632

[ref18] Federmeier K. D. Wlotko E. W. De Ochoa-Dewald E. Kutas M. (2007). Multiple effects of sentential constraint on word processing. Brain Res. 1146, 75–84. doi: 10.1016/j.brainres.2006.06.101, PMID: 16901469 PMC2704150

[ref19] Frisson S. Harvey D. R. Staub A. (2017). No prediction error cost in reading: evidence from eye movements. J. Mem. Lang. 95, 200–214. doi: 10.1016/j.jml.2017.04.007

[ref20] Grüter T. Rohde H. (2013). L2 processing is affected by RAGE: evidence from reference resolution. in 12th conference on Generative Approaches to Second Language Acquisition (GASLA).

[ref21] Guo X. F. (2012). How the word frequency-predictability affect the fixation duration and land position in Chinese reading. Master’s thesis, Tianjin: Tianjin Normal University.

[ref22] Kaan E. (2014). Predictive sentence processing in L2 and L1: what is different? Linguis. Approach. Bilingualism 4, 257–282. doi: 10.1075/lab.4.2.05kaa

[ref23] Kaan E. Dallas A. C. Wijnen F. (2010). “Syntactic predictions in second-language sentence processing,” in Structure preserved. Festschrift in the honor of Jan Koster. eds. Zwart J.-W. de Vries M. (Amsterdam: John Benjamins), 207–213.

[ref24] Kutas M. DeLong K. A. Smith N. J. (2011). A look around at what lies ahead: Prediction and predictability in language processing. Oxford: Oxford Academic press.

[ref25] Kwon N. Sturt P. Liu P. (2017). Predicting semantic features in Chinese: evidence from ERPs. Cognition 166, 433–446. doi: 10.1016/j.cognition.2017.06.010, PMID: 28641220

[ref26] Li Y. (2016). A study on word skipping during Chinese sentence Reading: Effects of word length and predictability. Master’s thesis, Changsha: Hunan Normal University.

[ref27] Li L. Zhao S. N. Zhang L. J. Wang J. X. (2022). Understanding mechanisms of prediction error cost in Chinese reading for older adults. Adv. Psychol. Sci. 30, 1–14. doi: 10.3724/SP.J.1042.2022.00001

[ref28] Liu Z. F. Tong W. Zhang Z. J. Zhao Y. J. (2020). Predictability impacts word and character processing in Chinese reading: evidence from eye movements. Acta Psychol. Sin. 52, 1031–1047. doi: 10.3724/SP.J.1041.2020.01031

[ref29] Liu N. N. Wang X. Liu Z. F. Wang Y. S. Yan G. L. (2019). The effect of contextual predictability on Parafoveal process for highly- and low-skilled developing readers during Chinese Reading. J. Psychol. Sci. 4, 848–853. doi: 10.16719/j.cnki.1671-6981.20190412

[ref30] Luke S. G. Christianson K. (2016). Limits on lexical prediction during reading. Cogn. Psychol. 88, 22–60. doi: 10.1016/j.cogpsych.2016.06.002, PMID: 27376659

[ref31] Mitchell D. C. Green D. W. (1978). The effects of context and content on immediate processing in reading. Q. J. Exp. Psychol. 30, 609–636. doi: 10.1080/14640747808400689

[ref32] Moreno E. M. Federmeier K. D. Kutas M. (2002). Switching languages, switching palabras (words): an electrophysiological study of code switching. Brain Lang. 80, 188–207. doi: 10.1006/brln.2001.2588, PMID: 11827443

[ref33] Rayner K. Ashby J. Pollatsek A. Reichle E. D. (2004). The effects of frequency and predictability on eye fixations in reading: implications for the EZ reader model. J. Exp. Psychol. Hum. Percept. Perform. 30, 720–732. doi: 10.1037/0096-1523.30.4.720 15301620

[ref34] Rayner K. Li X. Juhasz B. J. Yan G. (2005). The effect of word predictability on the eye movements of Chinese readers. Psychon. Bull. Rev. 12, 1089–1093. doi: 10.3758/BF03206448, PMID: 16615333

[ref35] Rayner K. Reichle E. D. Stroud M. J. Williams C. C. Pollatsek A. (2006). The effect of word frequency, word predictability, and font difficulty on the eye movements of young and older readers. Psychol. Aging 21, 448–465. doi: 10.1037/0882-7974.21.3.448, PMID: 16953709

[ref36] Rayner K. Well A. D. (1996). Effects of contextual constraint on eye movements in reading: a further examination. Psychon. Bull. Rev. 3, 504–509. doi: 10.3758/BF03214555, PMID: 24213985

[ref37] Roland D. Yun H. Koenig J. P. Mauner G. (2012). Semantic similarity, predictability, and models of sentence processing. Cognition 122, 267–279. doi: 10.1016/j.cognition.2011.11.011 22197059

[ref38] Staub A. (2015). The effect of lexical predictability on eye movements in reading: critical review and theoretical interpretation. Lang. Linguis. Compass 9, 311–327. doi: 10.1111/lnc3.12151

[ref39] Van Berkum J. J. Brown C. M. Zwitserlood P. Kooijman V. Hagoort P. (2005). Anticipating upcoming words in discourse: evidence from ERPs and reading times. J. Exp. Psychol. Learn. Mem. Cogn. 31, 443–467. doi: 10.1037/0278-7393.31.3.443 15910130

[ref40] Wang X. (2017). The contextual facilitation effect for developing readers’ lexical processing in natural Reading: The evidence from eye movements, Master’s thesis, Tianjin: Tianjin Normal University.

[ref41] Wang L. H. Bai X. J. Yan G. L. Wu J. (2012). The role of word frequency and word predictability in reading of elderly people. Chin. J. Gerontol. 16, 3503–3507. doi: 10.3969/j.issn.1005-9202.2012.16.073

[ref42] Wicha N. Y. Moreno E. M. Kutas M. (2004). Anticipating words and their gender: an event-related brain potential study of semantic integration, gender expectancy, and gender agreement in Spanish sentence reading. J. Cogn. Neurosci. 16, 1272–1288. doi: 10.1162/0898929041920487, PMID: 15453979 PMC3380438

[ref43] Zhang M. M. (2015). The mechanism of word skipping in Chinese Reading: An eye movement study. Doctoral dissertation, Tianjin: Tianjin Normal University.

[ref44] Zhang W. Chow W. Y. Liang B. Wang S. (2019). Robust effects of predictability across experimental contexts: evidence from event-related potentials. Neuropsychologia 134:107229. doi: 10.1016/j.neuropsychologia.2019.107229, PMID: 31610184

[ref45] Zhang W. Dong J. Duan X. Zhang Y. Gao X. Zhen A.. (2023). Prediction of semantic features is modulated by global prediction reliability: evidence from the N400 effect. J. Neurolinguistics 65:101109. doi: 10.1016/j.jneuroling.2022.101109

[ref46] Zhao S. N. (2009). Effect of word predictability on lexical processing in young and older Chinese readers: An eye movement study, Master’s thesis, Tianjin: Tianjin Normal University.

[ref47] Zhao S. W. (2020). Interaction effects of contextual predictiveness and parafoveal anticipation in children’s sentence reading, Master’s thesis, Tianjin: Tianjin Normal University.

[ref48] Zhao S. N. (2022). Mechanism of the predictive effect of context in sentence reading, Doctoral dissertation, Tianjin: Tianjin Normal University.

[ref49] Zhao S. N. Li L. Zhang L. J. Wang J. X. (2021). The influence of constrictive context on the processing of unpredictable word in Chinese Reading. Stud. Psychol. Behav. 19, 736–742.

[ref50] Zhu X. P. (1991). The effect of Chinese sentence context on word recognition. Acta Psychol. Sin. 23, 35–42.

[ref51] Zirnstein M. van Hell J. G. Kroll J. F. (2018). Cognitive control ability mediates prediction costs in monolinguals and bilinguals. Cognition 176, 87–106. doi: 10.1016/j.cognition.2018.03.001, PMID: 29549762 PMC5953823

